# Supplementary material for: Longitudinal Analysis of Urinary Cytokines and Biomarkers in COVID-19 Patients with Subclinical Acute Kidney Injury
Source: Int J Mol Sci. 2022 Dec 6;23(23):15419. doi: 10.3390/ijms232315419 (PMC9737068; doi:10.3390/ijms232315419)
Supplement: Supplementary file 1 [file ijms-23-15419-s001.zip › Supplementary Table S1. Imputation of cytokine and chemokine missing values.pdf]

Supplementary Table S1. Imputation of cytokine and chemokine missing values.

Cytokine or chemokine	Detectable / Missing* values (%)	Differences between complete / incomplete data				
		**Age, years (p)**	**Men, %/% (p)**	**SOFA, points (p)**	**AKI %/% (p)**	**Death %/% (p)**
FGF, Day 1	43/8 (15.6%)	53/55 (0.65)	58/63 (0.81)	4/6 (0.20)	53/63 (0.63)	23/0 (0.12)
FGF, Day 5	37/12 (24.4%)	52/60 (0.07)	54/45 (0.44)	4/3 (0.22)	48/66 (0.27)	18/16 (0.86)
IL-1β, Day 1	45/6 (11.7%)	53/60 (0.23)	60/50 (0.64)	3/6 (0.07)	56/50 (0.79)	20/13 (0.84)
IL-1β, Day 5	44/5 (10.2%)	53/70 (0.29)	56/60 (0.89)	3/6 (0.09)	50/80 (0.20)	18/20 (0.92)
G-CSF, Day 1	28/23 (45%)	47/56 **(0.02)**	68/48 (0.14)	3/6 (0.08)	54/57 (0.83)	18/22 (0.72)
G-CSF, Day 5	28/21 (42.8%)	52/53 (0.86)	64/47 (0.24)	3/4 (0.56)	46/61 (0.28)	25/9 (0.16)
IL-10, Day 1	19/32 (62.7%)	53/53 (0.64)	74/50 (0.09)	4/4 (0.64)	63/50 (0.36)	26/16 (0.35)
IL-10, Day 5	14/35 (71.4%)	55/52 (0.15)	64/54 (0.52)	4/4 (0.63)	64/48 (0.31)	7/22 (0.19)
IL-13, Day 1	41/10 (19.6%)	53/54 (0.31)	56/70 (0.42)	3/5 (0.23)	54/60 (0.71)	17/30 (0.35)
IL-13, Day 5	38/11 (22.4%)	51/56 (0.22)	55/63 (0.62)	4/4 (0.58)	47/72 (0.13)	15/27 (0.38)
IL-6, Day 1	51/0 (0%)	Complete data	Complete data	Complete data	Complete data	Complete data
IL-6, Day 5	48/1 (2.0%)	53/56 (0.67)	56/100 (0.38)	4/2 (0.21)	54/0 (0.28)	18/0 (0.63)
IL-12, Day 1	37/14 (27.4%)	53/54 (0.68)	54/71 (0.26)	4/3 (0.24)	57/50 (0.66)	19/21 (0.84)

IL-12, Day 5	33/16 (32.6%)	53/54 (0.84)	54/62 (0.59)	4/3 (0.21)	48/62 (0.35)	15/25 (0.40)
RANTES Day 1	29/22 (43.1%)	53/53 (0.84)	55/64 (0.54)	4/3 (0.19)	66/41 (0.08)	21/18 (0.82)
RANTES Day 5	23/26 (53.0%)	51/55 (0.14)	43/69 (0.06)	4/4 (0.40)	43/61 (0.20)	17/19 (0.86)
Eotaxin, Day 1	32/19 (37.2%)	53/53 (0.94)	59/58 (0.91)	3/4 (0.35)	50/63 (0.36)	22/16 (0.59)
Eotaxin, Day 5	30/19 (38.7%)	55/52 (0.32)	56/57 (0.93)	4/3 (0.46)	53/52 (0.96)	23/10 (0.25)
IL-17A, Day 1	22/29 (56.8%)	47/56 (0.08)	64/55 (0.54)	3/4 (0.19)	50/58 (0.54)	23/17 (0.62)
IL-17A, Day 5	23/26 (53.0%)	51/55 (0.11)	65/50 (0.28)	3/6 (0.11)	52/ 53 (0.90)	26/11 (0.18)
MIP-1α, Day 1	35/16 (31.3%)	53/54 (0.52)	60/56 (0.80)	3/4 (0.58)	51/62 (0.46)	23/13 (0.38)
MIP-1α, Day 5	21/28 (57.1%)	54/52 (0.39)	52/60 (0.56)	4/4 (0.64)	57/50 (0.62)	19/17 (0.91)
GM-CSF, Day 1	51/0 (0%)	Complete data	Complete data	Complete data	Complete data	Complete data
GM-CSF, Day 5	49/0 (0%)	Complete data	Complete data	Complete data	Complete data	Complete data
MIP-1β, Day 1	48/3 (5.8%)	53/53 (0.84)	60/33 (0.35)	4/2 (0.08)	54/67 (0.67)	19/33 (0.53)
MIP-1β, Day 5	48/1 (2.0%)	53/73 (0.10)	56/100 (0.38)	4/6 (0.42)	52/100 (0.34)	18/0 (0.63)
MCP-1, Day 1	51/0 (0%)	Complete data	Complete data	Complete data	Complete data	Complete data
MCP-1, Day 5	49/0 (0%)	-	-	-	-	-
IL-15, Day 1	37/14 (27.4%)	54/45 (0.14)	62/50 (0.43)	4/4 (0.64)	57/50 (0.66)	24/7 (0.16)
IL-15, Day 5	25/24 (48.9%)	54/53 (0.52)	64/50 (0.32)	4/3 (0.27)	64/4 (0.11)	20/16 (0.76)

EGF, Day 1	26/25 (49%)	45/56 (0.06)	65/52 (0.33)	3/4 (0.81)	58/52 (0.68)	23/16 (0.52)
EGF, Day 5	27/22 (44.8%)	53/53 (0.38)	70/40 **(0.03)**	3/4 (0.78)	59/45 (0.33)	25/9 (0.13)
IL-5, Day 1	31/20 (39.2%)	51/54 (0.25)	55/65 (0.47)	4/4 (0.36)	55/55 (0.99)	16/25 (0.43)
IL-5, Day 5	24/25 (51.0%)	46/57 **(0.01)**	50/64 (0.32)	3/4 (0.65)	54/52 (0.87)	20/16 (0.66)
HGF, Day 1	45/6 (11.7%)	53/53 (0.77)	56/83 (0.19)	4/2 (0.36)	53/67 (0.53)	20/16 (0.84)
HGF, Day 5	37/12 (24.4%)	53/53 (0.20)	56/58 (0.92)	4/3 (0.17)	56/41 (0.36)	18/16 (0.86)
VEGF, Day 1	48/3 (5.8%)	53/53 (0.52)	58/67 (0.77)	4/4 (0.82)	54/67 (0.67)	21/0 (0.37)
VEGF, Day 5	49/0 (0%)	-	-	-	-	-
IFN-γ, Day 1	34/17 (33.3%)	53/56 (0.21)	53/71 (0.22)	4/4 (0.79)	50/65 (0.32)	21/18 (0.80)
IFN-γ, Day 5	32/17 (34.6%)	52/56 (0.20)	50/70 (0.16)	3/4 (0.92)	46/64 (0.23)	18/17 (0.92)
IFN-α, Day 1	49/2 (3.9%)	53/55 (0.69)	57/100 (0.22)	4/5 (0.53)	53/100 (0.19)	20/0 (0.47)
IFN-α, Day 5	47/2 (4.0%)	53/47 (0.61)	55/100 (0.21)	4/2 (0.07)	53/50 (0.92)	19/0 (0.49)
IL-1Rα, Day 1	46/5 (9.8%)	53/40 (0.47)	63/20 (0.06)	4/3 (0.07)	59/20 (0.09)	20/20 (0.98)
IL-1Rα, Day 5	46/3 (6.1%)	53/39 (0.13)	58/33 (0.39)	4/3 (0.89)	52/66 (0.62)	17/33 (0.49)
TNF-α, Day 1	18/33 (64.7%)	42/56 **(0.02)**	61/58 (0.80)	3/4 (0.27)	44/61 (0.26)	22/18 (0.72)
TNF-α, Day 5	18/31 (63.2%)	42/56 **(0.03)**	61/ 54 (0.66)	3/4 (0.37)	44/58 (0.35)	22/16 (0.59)
IL-2, Day 1	36/15 (29.4%)	52/56 (0.10)	50/80 **(0.04)**	4/4 (0.53)	53/60 (0.63)	22/13 (0.46)

IL-2, Day 5	30/19 (38.7%)	53/53 (0.43)	46/73 (0.06)	3/4 (0.97)	46/63 (0.26)	20/15 (0.71)
IL-7, Day 1	48/3 (5.8%)	53/53 (0.65)	54/67 (0.77)	4/6 (0.52)	28/0 **(0.04)**	21/0 (0.37)
IL-7, Day 5	39/10 (20.4%)	53/54 (0.24)	56/60 (0.83)	4/4 (0.66)	51/60 (0.62)	15/30 (0.28)
IP-10, Day 1	28/23 (45%)	52/54 (0.23)	64/52 (0.38)	3/4 (0.99)	54/57 (0.83)	11/30 (0.07)
IP-10, Day 5	23/26 (53.0%)	51/54 (0.16)	56/57 (0.93)	3/4 (0.92)	52/53 (0.90)	30/7 **(0.04)**
IL-2R, Day 1	49/2 (3.9%)	53/54 (0.90)	61/0 (0.08)	4/5 (0.56)	53/100 (0.19)	20/0 (0.47)
IL-2R, Day 5	45/4 (8.1%)	53/66 (0.12)	57/50 (0.76)	4/4 (0.94)	48/100 (0.05)	20/0 (0.32)
MIG, Day 1	43/8 (15.6%)	53/58 (0.69)	61/50 (0.58)	4/5 (0.79)	54/63 (0.63)	21/12 (0.58)
MIG, Day 5	39/10 (20.4%)	53/52 (0.71)	61/40 (0.21)	4/4 (0.65)	56/40 (0.35)	17/20 (0.88)
IL-4, Day 1	3/48 (94.1%)	42/53 (0.47)	33/60 (0.35)	3/4 (0.58)	100/52 (0.10)	0/20 (0.37)
IL-4, Day 5	3/46 (93.8%)	51/53 (0.96)	66/56 (0.73)	4/4 (0.73)	66/52 (0.62)	33/17 (0.49)
IL-8, Day 1	48/3 (5.8%)	53/39 (0.07)	60/33 (0.35)	4/3 (0.58)	54/67 (0.67)	20/0 (0.37)
IL-8, Day 5	48/1 (2.0%)	53/51 (0.75)	56/100 (0.38)	4/2 (0.21)	54/0 (0.28)	18/0 (0.63)

Data are expressed as medians. Comparisons between complete and incomplete data were made using Mann-Whitney U for continuous variables.

Bold values denote statistical significance at the $p \leq 0.05$ level.

Acute kidney injury (AKI); fibroblast growth factor (FGF); granulocyte colony-stimulating factor (G-CSF); granulocyte/macrophage-colony stimulating factor (GM-CSF); RANTES (regulated on activation, normal T cell expressed and secreted); macrophage inflammatory protein (MIP); monocyte chemoattractant protein-1 (MCP-1); epidermal growth factor (EGF); hepatocyte growth factor (HGF); vascular endothelial growth factor (VEGF); interferon alpha (IFN-α); interferon-gamma-inducible protein 10 (IP-10); monokine induced by IFN-γ (MIG); interleukin (IL); IL-1 receptor (IL-1R).

Day 1: day of admission to critical care areas; Day 5: the fifth day in critical care areas.

*Missing values were those outside the ranges specified by the Luminex manufacturer. Missing values were imputed according to a practical guide for multiple imputation of missing data in nephrology [41].
