# Supplementary material for: Longitudinal Analysis of Urinary Cytokines and Biomarkers in COVID-19 Patients with Subclinical Acute Kidney Injury
Source: Int J Mol Sci. 2022 Dec 6;23(23):15419. doi: 10.3390/ijms232315419 (PMC9737068; doi:10.3390/ijms232315419)
Supplement: Supplementary file 1 [file ijms-23-15419-s001.zip › Supplementary Table S2. Principal component analysis of cytokines.pdf]

Supplementary Table S2. Principal component analysis of cytokines.

Component	Eigen value	Variance explained	Accumulated variance	Component weight
1	**6.48**	23.17	23.17	IFN-α (**9.1**)<br>EGF (**8.9**)<br>IL-13 (6.9)<br>VEGF (6.13)<br>Eotaxin (5.85)
2	**4.82**	17.21	40.38	IL-1R (**13**)<br>G-CSF (**10.7**)<br>IP-10 (**10.4**)<br>IL-5 (**9.6**)<br>RANTES (6.6)
3	**3.88**	13.88	54.27	IL-10 (**11.2**)<br>IL 15 (7.65)<br>IL-2R (6.95)<br>IL-4 (6.54)<br>IL-7 (5.6)
4	**3.27**	11.70	65.97	IL-12 (**8.8**)<br>MIP-1β (**8.4**)<br>IL-8 (7.1)<br>IL-17A (6.3)<br>IL-1β (6.3)

5	**2.08**	7.46	73.43	HGF (**23**)<br>MCP-1 (**11.8**)<br>IL-6 (**10.3**)<br>GM-CSF (6.4)
6	1.25	4.46	77.90	NA
7	0.88	3.16	81.07	NA

The principal component analysis retained components with eigenvalue >2 and explained >70% of the accumulated variance [42].

Components with eigenvalues >2 are in bold.

Cytokines with a component weight >8 are in bold.

Interferon alpha (IFN-α); epidermal growth factor (EGF); interleukin (IL); vascular endothelial growth factor (VEGF); IL-1 receptor (IL-1R); granulocyte colony-stimulating factor (G-CSF); interferon-gamma-inducible protein 10 (IP-10); RANTES (regulated on activation, normal T cell expressed and secreted); macrophage inflammatory protein (MIP)-1β; hepatocyte growth factor (HGF); monocyte chemoattractant protein-1 (MCP-1); granulocyte/macrophage-colony stimulating factor (GM-CSF).
